# Data-Driven Management—A Dynamic Occupancy Approach to Enhanced Rabies Surveillance Prioritization

**DOI:** 10.3390/v13091795

**Published:** 2021-09-09

**Authors:** Amy J. Davis, Jordona D. Kirby, Richard B. Chipman, Kathleen M. Nelson, Amy T. Gilbert

**Affiliations:** 1 National Wildlife Research Center, Wildlife Services, Animal and Plant Health Inspection Service, United States Department of Agriculture, Fort Collins, CO 80521, USA; amy.t.gilbert@usda.gov; 2 National Rabies Management Program, Wildlife Services, Animal and Plant Health Inspection Service, United States Department of Agriculture, Concord, NH 03301, USA; jordona.d.kirby@usda.gov (J.D.K.); richard.b.chipman@usda.gov (R.B.C.); kathleen.m.nelson@usda.gov (K.M.N.)

**Keywords:** detection probability, dynamic occupancy, rabies lyssavirus, raccoon, science-based management, surveillance, wildlife disease

## Abstract

Rabies lyssavirus (RABV) is enzootic in raccoons across the eastern United States. Intensive management of RABV by oral rabies vaccination (ORV) has prevented its spread westward and shown evidence of local elimination in raccoon populations of the northeastern US. The USDA, Wildlife Services, National Rabies Management Program (NRMP) collaborates with other agencies to implement broad-scale ORV and conducts extensive monitoring to measure the effectiveness of the management. Enhanced Rabies Surveillance (ERS) was initiated during 2005 and updated in 2016 to direct surveillance efforts toward higher-value specimens by assigning points to different methods of encountering specimens for collection (strange-acting, roadkill, surveillance-trapped, etc.; specimen point values ranged from 1 to 15). We used the 2016–2019 data to re-evaluate the point values using a dynamic occupancy model. Additionally, we used ERS data from 2012–2015 and 2016–2019 to examine the impact that the point system had on surveillance data. Implementation of a point system increased positivity rates among specimens by 64%, indicating a substantial increase in the efficiency of the ERS to detect wildlife rabies. Our re-evaluation found that most points accurately reflect the value of the surveillance specimens. The notable exception was that samples from animals found dead were considerably more valuable for rabies detection than originally considered (original points = 5, new points = 20). This work demonstrates how specimen prioritization strategies can be used to refine and improve ERS in support of wildlife rabies management.

## 1. Introduction

Effective surveillance is a fundamental component of wildlife disease management. Understanding the spatial and temporal extent of disease occurrence is critical to enact appropriate management actions aimed at the prevention of disease spread and elimination [1]. Surveillance of wildlife diseases can be particularly challenging as wildlife are evasive, the probability of encountering a diseased animal may vary with surveillance method, and not all diseased animals may be targeted for testing, particularly when a wildlife disease is not known to be zoonotic. Resources to conduct disease surveillance targeting wildlife are often limited [2]; therefore, it is important to understand how to maximize the probability of detecting diseases in wildlife populations through enhanced surveillance efforts.

A recognized wildlife zoonosis of global importance is rabies lyssavirus (RABV). Multiple variants of RABV independently and naturally circulate in carnivore and bat wildlife populations [3]. In the United States, raccoons (*Procyon lotor*) constitute the largest proportion of rabid terrestrial wildlife and are most frequently infected with the raccoon variant of RABV [4]. The World Organisation for Animal Health (OIE) supports controlling rabies in source populations, such as in raccoons in North America [5]. Oral rabies vaccination (ORV) is a management approach that has proven effective to control red fox (*Vulpes vulpes*) RABV in Western and Central Europe, canine RABV in coyotes (*Canis latrans*) in the United States, and raccoon RABV in Canada [6,7,8]. The raccoon RABV variant has been actively managed using ORV along the eastern coast of the United States since the 1990s [9].

Surveillance is critical to know the spatial extent of the problem prior to management, the spread of disease to new areas or populations, and for assessments of disease elimination across heterogeneous landscapes [10]. Public health surveillance is the primary source for describing trends in wildlife RABV circulation in the United States, which is focused on managing and mitigating risks of human and domestic animal exposures to rabid wildlife. The United States Department of Agriculture (USDA), Animal and Plant Health Inspection Service (APHIS), Wildlife Services (WS), National Rabies Management Program (NRMP; hereafter WS) instituted an enhanced rabies surveillance (ERS) program in 2004 to complement public health surveillance in support of management targeting raccoon RABV [11]. The WS ERS represents an active, targeted method to address specific management needs (e.g., RABV prevalence and/or spread in target wildlife populations).

ERS can be comprised of multiple methods of surveillance including reports of strange acting animals (hereafter, sample category strange-acting), animals that were found dead upon encounter (but not along roads and with no obvious explanation; hereafter, found dead), animals found as roadkill from formal survey methods or opportunistic sampling (hereafter, roadkill), animals trapped specifically for RABV surveillance (hereafter, surveillance-trapped), animals that were nuisance-trapped or reported by the public as a nuisance but healthy (hereafter, NWCO/Other), and animals for which the fate was not known or could not be described to any aforementioned categories (hereafter, unknown). During 2004–2015, WS recognized that different surveillance specimens were more likely than others to detect RABV and began to develop a prioritization scheme for specimen collection to reflect these differences. In 2016, WS implemented a stratified point value system to give higher weights to specimen types that reflected the relative value of different surveillance methods [11] (Table 1). The updated ERS system ensured that specimens were categorized upon collection to one of the six methods and structured to increase the collection of high-value specimens across space and time by setting cumulative point target thresholds for WS ERS. This point system was designed to be refined and enhanced as new data became available.

**Figure 1:**
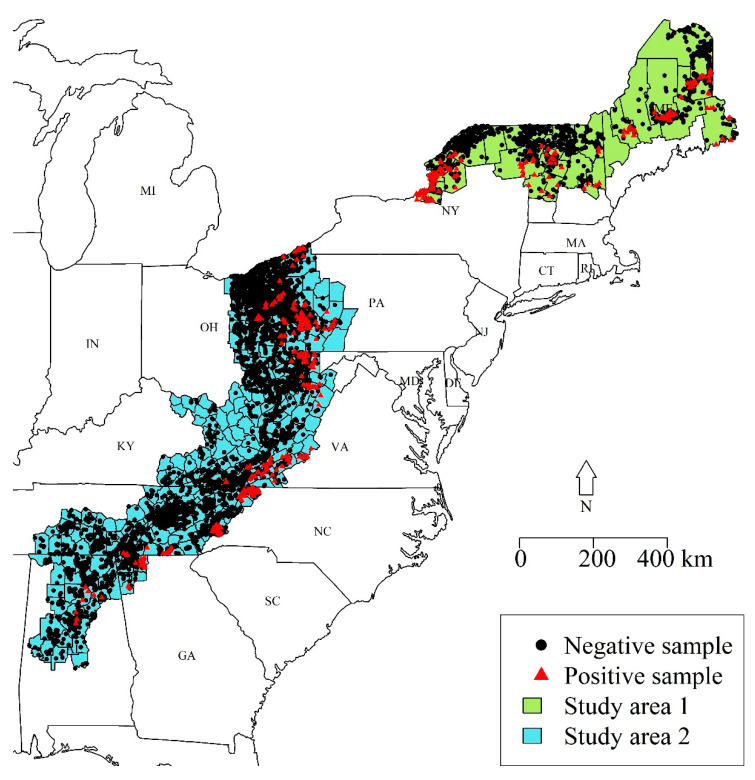
Map of counties from which the point-system re-evaluation was conducted. The analyses are conducted separately for two regions, study area 1, the northeastern region (light green), and study area 2, the lower east region (light blue). The infection status of specimens collected during 2016–2019 was evaluated using a direct rapid immunohistochemical test. Locations with rabies-negative raccoons are shown with black dots and rabid raccoons are shown with red triangles.

The points associated with each surveillance method were developed based on an expert review of ERS data collected from Ohio during 2005–2007 [11]. The objective of this study was to re-evaluate the point system using data from 2016–2019 and from across all US states with active ORV management (Figure 1) and to evaluate the impact of the implementation of the point system on the efficiency of RABV surveillance. The relative value of the different surveillance methods reflects the probability of detecting RABV when RABV is known to be present in an area. Therefore, we used a dynamic occupancy model, which estimates RABV occurrence across space and time, and simultaneously evaluates the probability of detection by the method of specimen encounter or collection. We sought to update the point system by using the detection probabilities across specimen types and rescaled the detection probabilities to the point-system scale.

## 2. Methods

We used ERS data from raccoon specimens collected by WS during 2016–2019 across all counties within two study areas of the eastern US (Figure 1). We used two geographically distinct study areas because there was a clear gap in ERS effort within the state of New York. Study area 1 included the northern parts of New York, Vermont, New Hampshire, and Maine (Figure 1). This region was dominated by deciduous and mixed forests (52.6%), evergreen forests (19.8%), and wetlands (10.3%). This study area bordered the St. Lawrence River and Quebec, Canada to the north and included Lake Champlain. The elevation ranged from sea level to 1710 m above sea level. Study area 2 included counties within the high-priority ERS areas from Pennsylvania to Alabama (Figure 1). This region was comprised of deciduous and mixed forest (60.9%), hay and pasture (15.3%), and open to low-intensity developed areas (9.4%). The elevation of study area 2 ranged from near sea level to 1891 m above sea level.

The WS ERS surveillance data included: date of specimen collection, species, location (latitude and longitude), surveillance method, and RABV status (positive or negative). RABV status testing was conducted by testing brainstem tissue samples collected from each animal using a direct rapid immunohistochemical test (dRIT; [12]). We used a multi-method dynamic occupancy model to estimate rabies occurrence (probability of rabies being present within a grid cell within a season) and detection probability (probability of rabies being detected given rabies was present) by surveillance method simultaneously for each study area as described previously [13]. We conducted separate analyses for the two regions as spatial gaps can bias RABV occurrence and detection estimates. Within each study region, we overlayed a 10 km by 10 km grid to evaluate the data using a resolution that matched WS ERS planning and effort. To account for variable incubation periods (e.g., 3–12 weeks) [14], we aggregated data by seasons (specifically astronomical seasons). Individual raccoons sampled within each season, within a grid constituted our secondary sampling, where a grid cell in a given season either was infected with raccoon RABV or was not infected. When a grid was known to be infected by at least one WS ERS method, the probability of detection for each method could be estimated using a multi-method approach [15]. To account for variations in RABV occurrence that may be related to raccoon abundance across heterogeneous landscapes, we allowed raccoon RABV occurrence to vary with habitat cover, elevation, season, ORV management status (indicating if the areas were managed with ORV or not in a given year), and temporal trends. This dynamic occupancy analysis was conducted using a Bayesian hierarchical model with a custom Markov Chain Monte Carlo algorithm with Metropolis–Hastings steps coded in program R [13,16,17].

In this analysis, raccoon RABV occurrence was not of primary interest; instead, we needed to adequately account for the variations in RABV occurrence to ensure estimates of detection by the WS ERS method were appropriately assessed. Detection probabilities only varied by the WS ERS method within a study area, as the point-system used by managers is currently implemented uniformly across season and location. Once estimates of RABV detection were estimated in each study area, we used a weighted average to estimate the detection probability across the combined study areas. We used a weighted average because the number of specimens collected within study area 1 was roughly one-sixth of the collection from study area 2. To derive the estimated points from this analysis, we set the lowest probability of detection to one integer point and divided each detection probability by this lowest value to get relative point values. We estimated the upper and lower 95% credible intervals for these points by similarly adjusting the 95% credible intervals for their detection estimates.

We also compared the number of specimens by WS ERS category collected during the four years prior to implementation of the point-system (2012–2015) against the four years of ERS points-system implementation (2016–2019). The WS ERS categories were not directly classified in the field prior to 2016. For the 2012–2015 data, we determined the surveillance categories post-hoc using the method of collection, the fate data, and the comments [13]. We compared the raw numbers of samples collected and the cumulative point totals as a measure of quality for those time periods.

## 3. Results

During 2016–2019, there were 20,488 raccoons sampled across both study areas, with 2849 raccoons sampled in study area 1 and 17,639 raccoons sampled in study area 2 (Table 2). There were 401 rabid raccoons across both study areas (127 from study area 1 and 274 from study area 2, Table 2). The majority of specimens from both study areas represent the NWCO/Other category (44.2% in study area 1 compared to 49.1% in study area 2). Roadkill was the second most common WS ERS method in study area 1 and the third most common method in study area 2 (39.5% and 15.6%, respectively). Strange-acting was the third most common method in study area 1 and the second most common method in study area 2 (11.7% and 22.0%, respectively).

**Figure 2:**
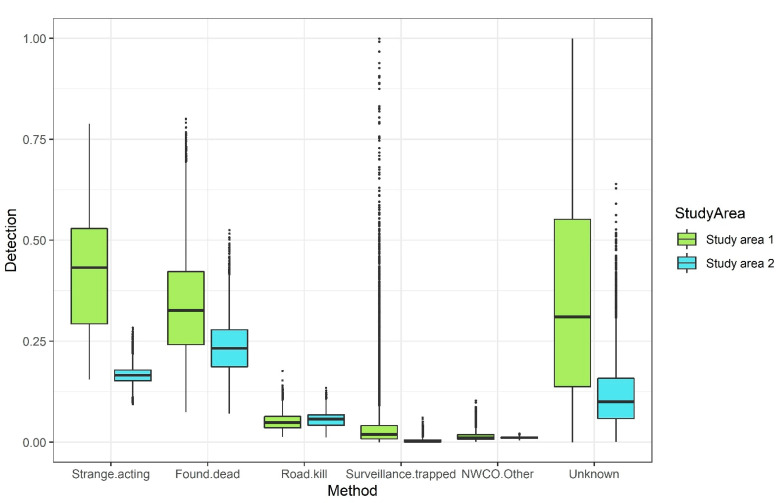
Estimated distributions of the detection probabilities by surveillance method and study area during 2016–2019 WS Enhanced Rabies Surveillance.

Animals reported to be strange-acting or found dead had the highest RABV detection probabilities between study areas (Figure 2). Animals reported with unknown status were associated with a high RABV detection probability but with considerable estimate uncertainty, which was reflective of the low sample sizes from that category (Figure 2 and Table 2). Animals collected as roadkill had the next highest detection rate in both study areas (Figure 2). Lastly, animals reported to be surveillance trapped and NWCO/Other categories had the lowest detection probabilities in both study areas (Figure 2).

The number of specimens collected from study area 1 was one-sixth of that from study area 2 (Table 2). The sample sizes per WS ERS method were used in the weighted averages to calculate the combined detection probabilities (Table 3). The new points across WS ERS methods were estimated by scaling the detection probabilities to match the scale of the current point system. This was carried out by dividing all values by the estimate for the NWCO/Other, thus setting this value to a point equal to one whole integer value. This scaling worked well, as the majority of the newly estimated points were within one integer of the current points.

To examine the impact of the point system on the ERS data collection, we compared the composition of samples four years prior to and four years following implementation. The number of samples across the two study regions increased from 15,938 to 20,488—a 29% increase in the four years after the ERS points-system implementation (Table 4). The cumulative points associated with WS ERS increased by 114% during that same time period, going from 42,603 points to 90,899 points (Table 4). Thus, the value of the samples increased considerably more than the number of samples post-implementation, suggesting that the point system helped focus surveillance efforts on higher-value samples as intended. The specimen raw positivity rates (the number of rabies positives divided by the total number of specimens tested) increased post-implementation from 1.5% to 2.0%. This was a statistically significant increase in raw positivity rate (*p*-value = 0.001 from a two-sample comparison of proportions). For comparison, the re-scaled points corresponded to fewer cumulative points pre- (37,908) compared to post-implementation (94,503).

## 4. Discussion

The WS Enhanced Rabies Surveillance (ERS) strategy was designed to increase the sampling intensity and to broaden the geographic scope for raccoon RABV detection [11]. From its inception, this strategy has been modified and refined to adapt to new information and management needs. The greatest change to this strategy was implemented during 2016 with the formalization of new best management practices and the stratified point-system guiding specimen collection. The difference we observed in the cumulative points associated with samples prior to and after the 2016 update demonstrates improved efficiency of the stratified point system for the detection of raccoon RABV. While the increase in the number of specimens collected and testing during this period increased points, there was only a 29% increase in specimens compared to a 114% increase in points, suggesting an increased focus on surveillance methods of higher value to WS raccoon RABV management. This improvement in surveillance quality was also demonstrated by a higher positivity rate post-implementation.

The methods available to observe natural phenomena (the observation process) often obscures the underlying ecological process of interest and can result in biased estimates of the ecology process or incorrect inference regarding ecological and/or epizootiological dynamics. In particular, wildlife disease epizootiological processes often have several layers of observation obscuring the underlying disease pattern; there is the observation of the host species and separately the observation of the wildlife disease. This multi-layered complexity can make surveillance of wildlife diseases particularly difficult for inference across broad and heterogeneous landscapes. Approaches that can improve our detection of wildlife disease can improve our understanding of spatial and temporal epizootiology. A 0.5 percentage point increase in raw RABV positivity among specimens may not seem substantial, but when working with a multi-state, multi-agency program charged with eliminating a deadly zoonosis, that improvement in efficiency can facilitate resource allocation to other program areas such as ORV management or monitoring efforts, with a goal towards raccoon RABV elimination.

It is important to note that the point values pre- and post-2016 are not directly comparable, as the field classifications were not directly assigned prior to 2016. Prior to 2016, classification of the specimen types was determined using a combination of data including specimen source, the fate of the animal, location of the animal, and the comments recorded upon collection [13]. There were considerably more samples classified post-hoc as “unknown” in the data prior to 2016, and this surveillance type only has a point value of one integer. Despite this, most animals that were strange-acting, found dead, or roadkill were able to be classified post-hoc using the information records, and thus the majority of these unknown samples likely would have fit into the NWCO/Other category (with the same point value). Therefore, even though the points may not be exactly the same had there been classifications in the field during 2012–2015, the proportion of samples that came from the highest point-value surveillance methods (strange acting, found dead, and roadkill) increased after the implementation of the program, suggesting real programmatic quality improvement.

The implementation of the ERS point-system has improved the overall probability of detecting raccoon RABV, which is critical to improving the efficiency of the program and informs the spatial and temporal distribution of raccoon RABV on the landscape [18]. The raw rate of positivity is not necessarily related directly to the probability of detecting a disease using that method [19]. The probability of detecting disease is conditioned on the disease being present (i.e., when the disease is absent there is no probability of detecting that disease; [20]). Raw positivity rates do not adjust for the presence of the disease and therefore generally underestimate the probability of detection. It is important to accurately estimate detection probabilities as these are used to determine the probability of disease elimination and evaluate whether surveillance efforts need refinement. Knowing when to refine program management or surveillance efforts entails substantial financial costs and accuracy is paramount. Furthermore, the patterns of positivity rates may obscure the actual value of different surveillance approaches to detect the disease when not accounting for disease presence. Jennelle et al. [19] demonstrated apparent prevalence (positivity rates) can suggest spurious or even incorrect relationships of disease occurrence and that detection probabilities should be estimated in studies examining disease dynamics. Therefore, we used a model that jointly estimates detection probability and disease occurrence to re-evaluate the current point system for the detection of raccoon RABV.

The results of this study show that the current point system generally does a good job at reflecting the relative values of the different surveillance methods (Table 2). Strange-acting and found dead samples were previously recognized as the most valuable for detecting RABV [11]; these two surveillance types were also found as the most valuable in our study. However, our study suggests that the found dead samples were considerably more valuable than previously thought (i.e., the newly estimated point value of 20 compared to 5). RABV has a mortality rate approaching 100% once clinical signs appear [21]. Although RABV may not be a major source of mortality for raccoon populations [22,23,24], raccoons that are found dead (not including roadkill) may be more likely to be infected with RABV due to acute neurologic and motor deterioration among infected animals. Roadkill and NWCO/Other specimens were about as valuable as expected, suggesting those points may not need further refinement. The surveillance-trapped method, however, was not as valuable as originally thought in detecting raccoon RABV. This may be in part due to rationale in how and when surveillance trapping is conducted. Previous work has suggested that detection probabilities of RABV vary with season [13], and the timing of surveillance trapping may be limited to seasons with lower infection prevalence. Additionally, it is possible that rabid animals may be less likely to be attracted to a trap compared to uninfected animals. Even when rabid animals are detected through surveillance trapping, they represent a very small portion of the specimens collected by ERS. However, it may be useful to focus future research on how to improve detection probabilities when using surveillance-trapping efforts may be desirable for other management reasons (e.g., contingency action or breach of ORV zone).

Prior studies have described seasonal fluctuations in RABV incidence [25,26]. In addition, detection probabilities [13] and positivity rates [11] for raccoon RABV were observed to have seasonal variations. Therefore, it is possible to have a point system that accounts for the fact that detection probabilities might be higher in some seasons than others within specimen types. Similarly, detection probabilities may vary by region or habitat and hypothetically points could vary with these elements as well. However, creating a complex temporally and spatially varying point-system has the potential to undermine the utility of an accessible and easy-to-understand approach that is practical for managers. Although some seasons may have higher detection probabilities than others, providing good temporal coverage is more important than targeting a single season with the highest incidence. This same approach can be used to further improve the ERS guidance by encouraging more even spatial distribution of specimen collections across and within political boundaries.

## 5. Conclusions

The implementation of the ERS point system has demonstrated a marked improvement in the detection probability of raccoon RABV which in turn has facilitated a greater understanding of management effectiveness. Following the principles of science-based adaptive management, the point system has been reevaluated and updated based directly on surveillance data collected by managers. The advantages of an ERS point system are that it remains flexible and can be updated based on future data and epizootiological trends. The primary goal of the WS NRMP is to eliminate raccoon RABV in the eastern US. As the ORV management areas are shifted through time, it will be beneficial to continue to ensure the point-system values are reflective of data for efficiency in management operations.

## Figures and Tables

**Table 1:** Enhanced Rabies Surveillance standardized categories and their relative values.

Category	Point Values	Description
Strange acting	15	Suspect behavior suggestive of neurological disease
Found dead	5	Unexplained with no obvious signs of trauma, not roadkill
Roadkill	3	Formal survey or opportunistic, 1 additional point/mile driven
Surveillance trapped	2	Active trapping in specified raccoon rabies risk areas/response to an outbreak
NWCO/Other	1	Nuisance-trapped or homeowner-derived; apparently healthy
Unknown	1	Behavior not observed; fate not determined

**Table 2:** Sample sizes and number of rabid raccoons during 2016–2019 in each study area and overall by surveillance method.

Surveillance Method	Overall		Study Area 1		Study Area 2	
	Sample Size	# Positive	Sample Size	# Positive	Sample Size	# Positive
Strange acting	4217	254	334	74	3883	180
Found dead	399	36	79	15	320	21
Roadkill	3871	64	1125	32	2746	32
Surveillance trapped	2035	0	51	0	1984	0
NWCO Other	9918	46	1258	6	8660	40
Unknown	48	1	2	0	46	1
Total	20,488	401	2849	127	17,639	274

**Table 3:** Enhanced Rabies Surveillance current and newly estimated points with 95% credible intervals (CI) by surveillance method. The model-averaged detection probabilities across study areas and 95% CIs are also provided by the method.

Surveillance Method	Current Points	Estimated Points	95% CI	Detection Probability	95% CI
Strange acting	15	14	(10, 18)	0.18	(0.13, 0.23)
Found dead	5	20	(11, 31)	0.25	(0.14, 0.39)
Roadkill	3	4	(2, 6)	0.05	(0.03, 0.07)
Surveillance trapped	2	1	(0, 2)	0.00	(0, 0.02)
NWCO/Other	1	1	(1, 2)	0.01	(0.01, 0.02)
Unknown	1	12	(1, 34)	0.16	(0.02, 0.43)

**Table 4:** Comparison of cumulative points based on sample sizes by surveillance method across four years prior to points-system implementation and the four years post-implementation across both study regions. The cumulative points are shown for the current point-system and the newly estimated point system.

	Sample Size		Cumulative Current Points		Cumulative Newly Estimated Points	
Surveillance Method	2012–2015	2016–2019	2012–2015	2016–2019	2012–2015	2016–2019
Strange acting	1379	4217	20,685	63,255	15,960	59,038
Found dead	188	399	940	1995	3220	7980
Roadkill	2192	3871	6576	11,613	7060	15,484
Surveillance trapped	2223	2035	4446	4070	1889	2035
NWCO/Other	5175	9918	5175	9918	5059	9918
Unknown	4781	48	4781	48	4720	48
Total	15,938	20,488	42,603	90,899	37,908	94,503

## Data Availability

Data associated with this paper have been deposited in the Research Data Archive.
